# Identification of Ancestry Informative Markers in Mediterranean Trout Populations of Molise (Italy): A Multi-Methodological Approach with Machine Learning

**DOI:** 10.3390/genes13081351

**Published:** 2022-07-28

**Authors:** Giovanna Salvatore, Valentino Palombo, Stefano Esposito, Nicolaia Iaffaldano, Mariasilvia D’Andrea

**Affiliations:** 1 Department of Agricultural, Environmental and Food Sciences, University of Molise, Via De Sanctis snc, 86100 Campobasso, Italy; g.salvatore5@studenti.unimol.it (G.S.); nicolaia@unimol.it (N.I.); dandrea@unimol.it (M.D.); 2 Mediterranean Trout Research Group, Via Porali 3, 42037 Ventasso, Italy; dott.stefanoesposito@gmail.com

**Keywords:** Mediterranean trout, introgression, SNP array, machine learning, random forest, ancestry informative markers

## Abstract

Brown trout (*Salmo trutta*), like many other freshwater species, is threated by the release in its natural environment of alien species and the restocking with allochthonous conspecific stocks. Many conservation projects are ongoing and several morphological and genetic tools have been proposed to support activities aimed to restore genetic integrity status of native populations. Nevertheless, due to the complexity of degree of introgression reached up after many generations of crossing, the use of dichotomous key and molecular markers, such as mtDNA, LDH-C1* and microsatellites, are often not sufficient to discriminate native and admixed specimens at individual level. Here we propose a reduced panel of ancestry-informative SNP markers (AIMs) to support on field activities for Mediterranean trout management and conservation purpose. Starting from the genotypes data obtained on specimens sampled in the main two Molise’s rivers (Central-Southern Italy), a 47 AIMs panel was identified and validated on simulated and real hybrid population datasets, mainly through a Machine Learning approach based on Random Forest classifier. The AIMs panel proposed may represent an interesting and cost-effective tool for monitoring the level of introgression between native and allochthonous trout population for conservation purpose and this methodology could be also applied in other species.

## 1. Introduction

The introduction of alien species is causing dramatic changes in many fresh water ecological systems worldwide, determining the erosion of local species integrity [1,2]. Among the others, brown trout (*S. trutta*) has traditionally attracted the attention of conservation biologists and public institutions due to its iconic significance for fishery management and aquaculture. Brown trout is considered a complex of incipient species [3,4], which counts several phylogenetic lineages including Mediterranean trout [5]. Recently, many conservation projects have been proposed to restore the genetic integrity status of Mediterranean trout populations in Italy [5,6,7,8,9,10], mainly threated by introgressive hybridization between native and commercial hatchery strains, often introduced for meeting fishing demands and for recreation activities. Morphological features (such as number of parr marks, adipose fin color pattern and number of black opercular spots [11]) have been successfully adopted in order to quickly differentiate among native, farm-reared and hybrid specimens during preliminary steps of monitoring activities [11] but the use of genetic markers remains pivotal to conduct a truly effective restoration project [12].

At a genetic level, the Mediterranean trout population can be easily discriminated from its Atlantic lineage through PCR-RFLP analysis of mtDNA segments in combination with nuclear LDH-C1* locus. Such loci have been extensively used in order to provide a rapid genetic characterization in many studies [8,9,13], sometimes in conjunction with the use of microsatellite markers to better assess the effectiveness of specific conservation objectives [14]. Nevertheless microsatellites suffer of lack replicability among laboratories, and not guarantee a large genome coverage [15] which is pivotal for the study of genetic structure at fine-scale. In this regard, recent advances in field of genomics resources and technologies represent key opportunities to overcome these issues and optimize conservation efforts in many wild species, including brown trout [16,17,18]. In particular, medium-density SNP arrays are now available for some salmonid species, such as rainbow trout and Atlantic salmon [19], and a recent large SNP array was developed also for brown trout [15]. Nevertheless, the *S. trutta* complex is one of the most genetically diverse vertebrate groups, consisting of more than 60 species, including several ecotypes and evolutionary lineages [20,21]. In this context, the development of species-specific array is still unrealistic; thus, the use of large SNP microarrays developed in one species to analyze a closely related species with limited genetic resources can be considered as an effective alternative [22]. In a recent work, Palombo et al. [6] successfully used for the first time the rainbow trout derived 57K SNP array [23] for the genetic characterization of two Mediterranean trout populations inhabiting Molise rivers (Central-Southern Italy). The authors reported useful information for a fine-scale genetic structure characterization and such results supported conservation and monitoring activities implemented by LIFE17 NAT/IT/000547 Project.

In order to provide a most affordable and cost-effective solution to further support native trout conservation and management activities we decided to exploit the genetic information obtained in such a previous study [6] to create a reduced SNP panel containing the most ancestry-informative markers (AIMs) with very little loss of information compared to initial Axiom 57K array genotyping solution. Several studies have shown that a reduced set of selected informative markers can effectively capture the genetic structure of populations in human and livestock [24,25,26] and several statistical analyses [25,26,27,28], as well as commercial tools [29], are reported to be helpful to this aim. Among the others, we decided to apply a Machine Learning (ML) approach, accordingly to what has been recently proposed by other authors [25,26,30], which used a Random Forest (RF) classifier to identify population informative SNPs useful for pig, cattle, and wild sheep breed identification.

## 2. Materials and Methods

### 2.1. Filtering Procedures and Reference Dataset Building

In total 288 specimens from Biferno and Volturno rivers, the main two basins of Molise, were enrolled in this study. As previously described [6], those samples were collected within LIFE17 NAT/IT/000547 Project’s activities and were genotyped with the rainbow trout Axiom SNP array [23], as well as screened by PCR-RFLP technique at *16S* rRNA and LDH-C1* loci, according to McMeel et al. [31] and Chiesa et al. [32]. Within such Project’s conservation activities, the combination of genotypes at *16S* rRNA and LDH-C1* loci has been used as criterion for the identification of six different classes of introgression (i.e., from class I ‘completely introgressed’ to class VI ‘no introgressed’), according to Pensierini et al. [33]. In particular, specimens of class VI have been declared as native and thus used for reproduction purpose during Project’s steps. Traditionally some authors have used different combinations of mitochondrial and nuclear genetic loci for genetic analysis of S. trutta [10,34,35,36].

An initial quality control (QC) of the dataset was performed by first applying a filter based on individual genotyping success and retaining profiles with ≥80% success rate. Next, data was screened with a per-SNP genotyping threshold of ≥95% and pruned for loci deviating Hardy–Weinberg equilibrium (HWE; *p*-value ≤ 10^−3^). SNPs that met the QC criteria were therefore used to perform a preliminary assessment of the entire dataset in Admixture software (v.1.3.0) [37] with K = 2 population cluster, roughly corresponding to native and alien trout population inhabiting Molise rivers, according to results reported by Palombo et al. [6]. Admixture analysis was performed considering each river separately to exclude possible hybrids. Non-admixed specimens, assigned with an admixture ancestry score (q_i_) ≥ 0.99 to their respective population clusters (i.e., native and alien populations), were retained to construct a reference dataset for AIMs identification.

### 2.2. Marker Selection

Four different statistical methods were employed on the dataset for marker information content estimation. Based on obtained results and considering that most common microplates have a 96-well plate format, the top 96 ranked SNPs were used to declare AIMs for each approach. Specifically, the first method, which has been one of the most popular for selecting informative loci, was the pairwise F_ST_ estimation of Weir and Cockerham [38] as calculated at each locus using PLINK software [39]. The second method relied on allele-frequency differential (Delta) estimation [40], which is one of the most straightforward ways to evaluate the information content of a SNP. In particular, for a bi-allelic marker, the Delta value is estimated as |pA_i_ − pA_j_|, where pA_i_ and pA_j_ are the frequencies of allele A in the ith and jth subpopulation. The Delta value for each SNP locus was estimated as the mean across all pair-wise comparisons. The third selection method was principal component analysis (PCA). Informative markers were selected, according to Paschou et al. [41], considering the sum of the squares of the most informative principal components (PC). The choice of the number of PC was determined by the amount of variance explained, as previously defined by Schiavo et al. [26], and the number of PC chosen in our analysis was three. The loadings for each SNPs were squared and summed over the most significant PC in order to produce an estimate of informativeness and finally used to rank SNPs. The fourth selection method was the RF classifier, which is a supervised ML algorithm based on an ensemble of decision trees. Implementation of the RF has been done using scikit-learn Python library [42]. RF algorithm measures the importance of a feature (i.e., a SNP) and evaluates the role of each feature in the classification that can be used as an indicator of SNP informativeness. SNPs were ranked using two different parameters implemented in criterion function: the Gini impurity (GI) and the Entropy (EN). Since different runs of the RF procedure can lead to slightly different results in terms of selected SNPs, 100 RF runs were performed. Finally, informative SNPs were selected based on two different procedures, according to Schiavo et al. [26]: (1) SNPs that occurred more frequently among the first top 96 SNPs list, after the 100 runs; (2) SNPs with the highest importance average value over the 100 runs. These two methods were applied to evaluate the stability of RF selection and leaded to two different candidate SNP panels for each RF criterion applied. Overall, seven panels of 96 SNPs were obtained through the different statistical approaches used for SNPs prioritization. Finally, shared SNPs among all different analyses were considered as the best candidate AIMs and, in turn, they were tested in a separated RF analysis.

### 2.3. Validation Dataset

In order to test and validate the AIM panels identified through marker selection analyses, a RF classifier was performed using genotype data from simulated hybrid populations. Simulated hybrids were artificially constructed using Hybridiser v0.1 R script developed by Somenzi et al. [30]. More in detail, a dataset of 60 simulated hybrids was generated for both rivers as follows: (i) 20 F1 offspring obtained by native x alien specimens, (ii) 20 backcrosses between F1 x alien specimens (BC1A) and (iii) 20 individuals obtained as backcross between F1 and native trout (BC1N). Although, as already observed by Schiavo et al. [26], RF does not need any cross-validation on a separate test set to get an unbiased estimate of the test set error, we decided to randomly split the validation dataset into a new reference (80% of specimens) and test population (remaining 20%). RF classifier was fitted on such new reference set and the corresponding out-of-bag (OOB) error score was calculated, which is an unbiased estimate of prediction accuracy. Classification performance was assessed also using the test population (i.e., animals not used to train the algorithm) and this allowed us to evaluate the fitted ML model.

Furthermore, to visually compare the performance of the full set of SNPs and the candidate AIMs shared among the seven panels, PCA was performed considering both simulated and real hybrid populations. Real hybrids were extracted from initial dataset considering a q_i_ admixture score <0.99. Finally, to measure how well the candidate AIMs estimated the admixture level compared to that determined by the full set of markers, we compared the admixture results using the coefficient of determination (r^2^). To test if the AIM panels performed better than an equally sized set of SNPs chosen at random, 1000 random AIM sets were generated, and for each random set supervised admixture analysis was performed. Finally, coefficients of determination values between the ancestry assignment of the full set and the reduced random panel were computed. The coefficient of determination values obtained using the 1000 random SNP sets were standardized by z-scores.

### 2.4. SNP Annotation

To further disentangle the information carried out by common AIMs identified across the seven reduced panels, 35 bp flanking sequence from each side of the SNP, provided by the array manufacturer, was aligned to *S. trutta* genome assembly (v. fSalTru1.1) by BLASTN software, considering an e-value cut-off of 1 × 10^−6^ and a percent identity threshold of the matching sequence ≥85%. Hits were used to infer position on the reference genome and annotate genes spanning a region of ±50 Kbp around each SNP using Ensembl Variant Effect Predictor (VEP) tool (release 107) [43]. In order to identify overrepresented terms in KEGG and GO knowledgebase, a pathway enrichment analysis was performed respectively by PANEV package [44] and by g:Profiler toolset [45], considering only annotated genes.

## 3. Results

### 3.1. Population Overview

**Figure 1:**
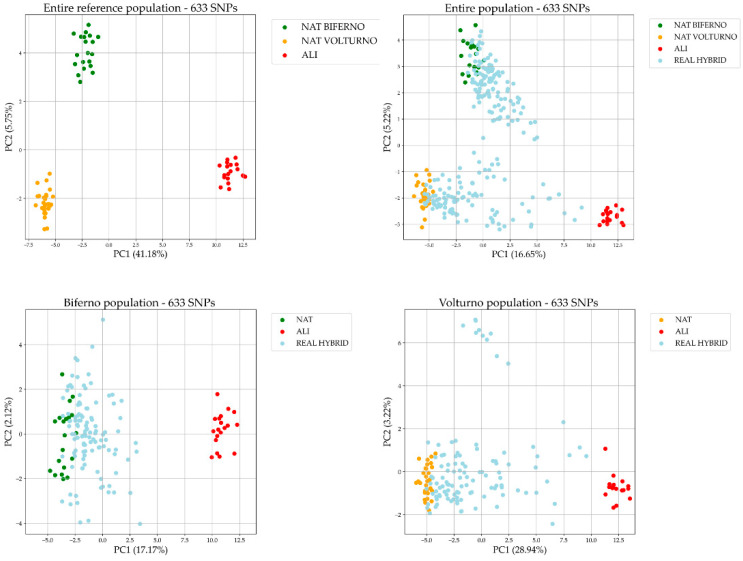
PCA obtained using the full SNPs set on reference and entire populations, encompassing non-admixed native and alien trout samples. In green and orange are reported the non-admixed native split by rivers and in red the alien samples, respectively. In brackets the percentage of variance explained by each component is reported.

In total, 633 SNPs and 288 specimens passed QC filtering. Considering admixture outcomes (q_i_ ≥ 0.99), 49 and 19 samples were classified as non-admixed native (NAT) or alien (ALI), respectively (Table S1) and were considered as reference population for SNP prioritization. PCA plot obtained using the 633 SNPs on entire reference population showed a clear separation of NAT and ALI samples in both rivers (Figure 1). PC1 (41.18% of total variance) split NAT and ALI trout as two distinct clusters whereas PC2 (5.75% of total variance) identified subpopulation structure among NAT trout of Biferno or Volturno rivers. As regarding introgression classes estimated through the combination of *16S* rRNA and LDH-C1* genotyping, the outcomes are reported in Table S1. The distribution of trout population ancestry scores for each introgression class, estimated with the combination of mtDNA and LDH-C1* genotypes [33], was reported in Figure S1, which suggested a heterogeneous scenario within each class. A preliminary PCA investigation on validation dataset composed by real hybrids was performed using the 633 SNPs and rerun on split by river datasets (Figure 1). PCA plots showed an admixed scenario caused by a significant hybridization level, in line with observations reported by Palombo et al. [6].

### 3.2. Comparison of AIMs Selection Methods and Validation

In total, seven different reduced panels were obtained, considering top 96 ranked SNPs selected by four different approaches applied on reference population. One panel was obtained by F_ST_, one by Delta, one by PCA statistics and four lists were derived using RF algorithm, applying GI and EN ranking methods (as described before, two stability procedures were tested for each applied methods). Table S2 reports the lists of top-96 SNPs detected by each method and included in the seven panels and Table 1 reports the number of shared AIMs between pairs of SNP panel determined with the seven different approaches.

In order to assess the reliability of the identified panels, a validation step was performed applying a RF classifier. OOB scores and correct prediction proportions are reported in Table 2. All samples were correctly assigned (100%) across all methods on training set (train accuracy) with an average OOB score of 88%. Focusing on testing set, test accuracy values were >92% across all approaches. The highest OOB score was detected for Delta method (91%), lowest for RF EN 1 (85%).

In total, 47 SNPs resulted in common among all top-ranked 96 SNP lists and therefore they were considered as the best candidate AIMs for the development of a reduced panel (Table S3). The RF classifier validation was performed also considering the 47 candidate AIMs. Performance outcomes were in line with expectations (OOB score 86%, train accuracy 100%, test accuracy 92%). The 47 common AIMs panel was also tested to detect admixture between native and alien specimens in both rivers. R^2^ values were high overall across all panels encompassing 96 SNPs (r^2^ ≥ 0.973; Table 3) and also the r^2^ calculated between the ancestry percentage obtained using 47 candidate AIMs and full set of SNP resulted quite high, i.e., 0.955 and 0.979 for Biferno and Volturno rivers, respectively (Table 3).

**Figure 2:**
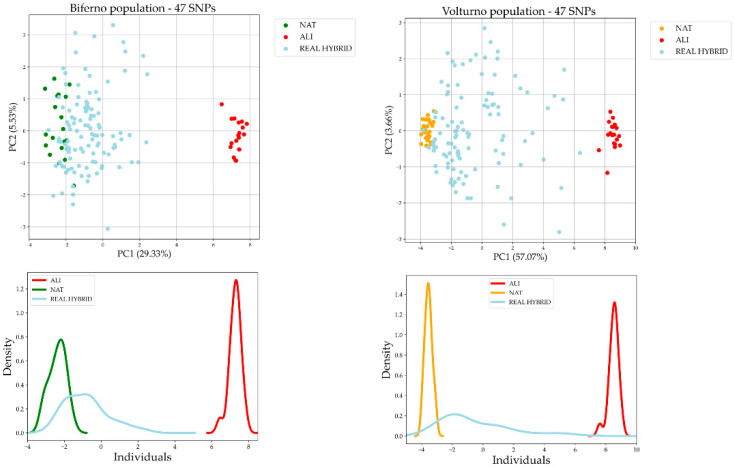
PCA and density distribution of the PC1 obtained using the common 47 AIMs on reference populations and real hybrid dataset split by rivers.

Furthermore, to visually compare the performance of the full set of SNPs to what obtained by 47 common AIMs, PCA was run considering both simulated and real hybrid populations for both rivers separately. Furthermore, OOB scores and correct prediction proportions on such data are reported in Table S4. Overall, PCA plots showed a clusterization comparable with the PCA results obtained by the full set of SNPs. Indeed, PCA of real hybrids (Figure 2) identified several individuals overlapping with the pure ancestry native cluster, while the others were distributed along a gradient between NAT and ALI.

**Figure 3:**
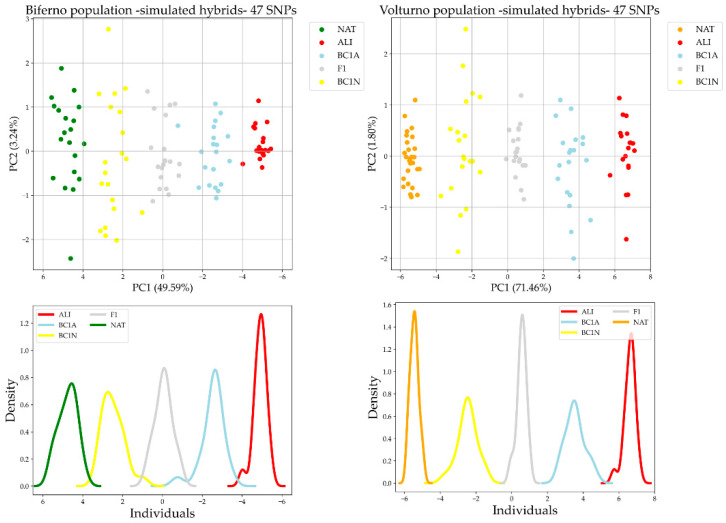
PCA and density distribution of the PC1 obtained using the common 47 AIMs on reference populations and simulated hybrid dataset split by rivers.

PCA on simulated hybrids (Figure 3) discriminated the parental populations (NAT and ALI) at opposite sides of the graph and positioned the hybrid populations according to their ancestry proportions, with F1 at the center of the plot and the two backcrosses BC1N and BC1A closer to NAT and ALI, respectively.

### 3.3. SNP Annotation and Marked Genes

**Figure 4:**
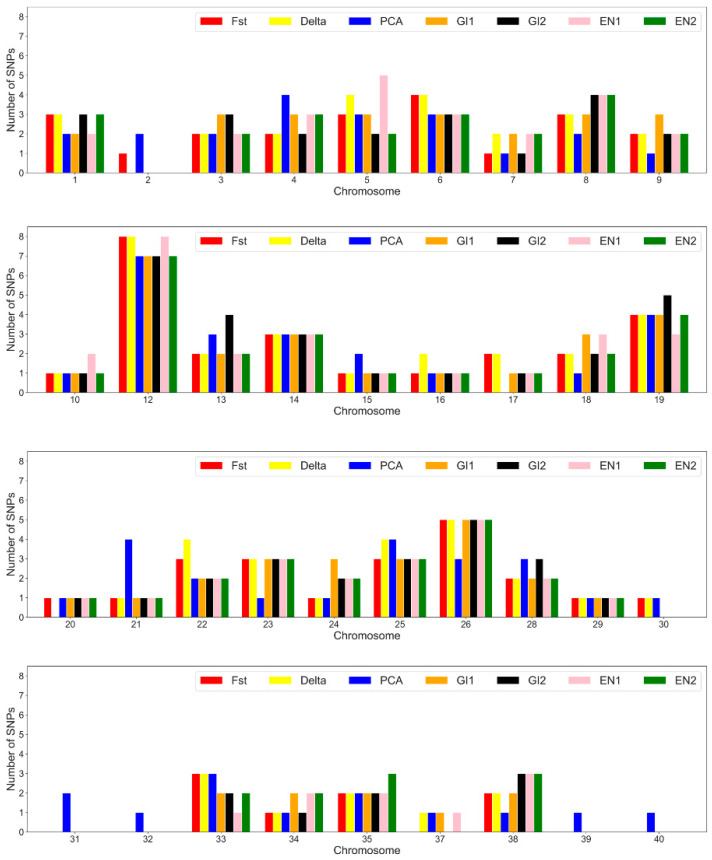
Distribution on the 40 trout chromosomes of the SNPs selected for the 96 SNP panels using the four different methods described in this study (RF GI 1 = random forest Gini Index stability occurrence; RF GI 2 = random forest Gini Index stability mean; RF EN 1 = random forest Entropy stability occurrence; RF EN 2 = random forest Entropy stability mean; Delta; F_ST_ = Fixation index; PCA = principal component analysis).

In total 466 out of 633 SNPs (~74%) were successfully mapped on *S. trutta* reference genome. Within panels, SNPs per chromosome ranged from 1 to 8. Considering all panels together, there was a similar distribution per chromosome of the selected SNPs. Highest number of top-ranked SNPs was harbored on chromosome 12, 19 and 26 (Figure 4). Focusing on 47 common AIMs, 41 out if 47 were mapped on *S. trutta* genome assembly and in total 143 genes were pinpointed by VEP tool [43], considering boundaries of 50 Kbp around each SNP (Table 4). No KEGG and GO terms were statistically significant overrepresented among our gene list.

## 4. Discussion

An increasing popularity of SNP analysis tool is widely recognizable in wild conservation projects to discriminate between pure and alien/hybrid specimens. However, the large-scale use of SNP arrays can be challenging for the average financial availability of conservation projects; thus, the development of a small panel of AIMs can be considered an effective alternative [25,26,30,46,47,48].

In this work, we evaluated marker selection methods and determine a small number of highly discriminant SNPs from the rainbow trout Axiom array, required to effectively and confidently assign individual genotypes to native and alien populations notably within LIFE17 NAT/IT/000547 Project’s activities. More in detail, the Project had the main goal to restore genetic integrity of native Mediterranean trout populations inhabiting Molise’s rivers. Our final aim is to support the monitoring and conservation activities proposed by the Project, through the development of a reduced SNP panel, which guaranties a rapid and low-cost genotyping analysis without significantly compromising its informativeness. Indeed, although the combination of mtDNA and LDH-C1* loci genotypes can be a useful approach to suggest the introgression degree at the population level, its consistency at individual level is far from being accurate, especially after several generations. Our results would corroborate this general consideration, indeed classes of introgression estimated by combination of mtDNA and LDH-C1* loci genotypes [33] were not always consistent with individual ancestry scores estimated by admixture (Figure S1). A clear concordance apparently was not detectable.

It is well-known that the accuracy of AIM panels depends on the quality and sample size of the reference populations. Clearly, a high number of genotyped samples helps to take into account the whole within population variability and, in turn, it reduces the possibility that few individuals might be not assigned correctly due to their atypical genotypes. Nevertheless, for many practical reasons it is not always possible to use large reference datasets. In our study, the number of specimens considered in the reference datasets was conditioned by Project objectives, which was focused on native trout conservation in Molise’s rivers. Furthermore, to the best of our knowledge, except for Palombo et al. study [6] there are no other available data using trout Axiom SNP array for Mediterranean trout populations’ characterization. The number of specimens considered in the final reference dataset of our study was 68 (i.e., 20 pure native Biferno trout, 29 pure native Volturno trout and 19 pure alien trout). Due to the large genetic distance occurring between Mediterranean and Atlantic trout lineages, we achieved reliable features selection using such sample size for each reference population and this is in line with what reported by Somenzei et al. [30].

A total of 633 SNPs was retained after filtering steps. PCA plots obtained using quality filtered SNP datasets showed a clear separation of native and alien trout populations in both rivers (Figure 1). Four statistical methods were used for the identification of informative SNP panels (i.e., Delta, F_ST_, PCA and RF statistics), according to Schiavo et al. [26]. Several approaches have been proposed in literature for the identification of population-informative markers [40] and it is known that the choice of a specific approach can affect the results for a particular population [49]. As explained by Bertolini et al. [50], the main problems for the identification of fully informative SNP markers are due by the high level of linkage disequilibrium (LD) that is present in most livestock populations. In this regard, it is significant to highlight that a supervised machine-learning-based classification approach has been demonstrated to be able to partially reduce this problem [25]. Furthermore, it is noteworthy to highlight that our study involved a wild population where the LD could be considered much less extensive compared to livestock species. Stability of RF selection was assessed implementing a method based on iterations and evaluating the frequencies by which SNPs were selected and the mean values of the ranking parameters as already proposed by other authors [26]. This leaded to two different candidate SNP panels for each RF criterion applied. Overall, seven different reduced panels including top ranked 96 AIMs each were selected by four approaches applied (Table 1). Four panels derived using RF by applying GI and EN ranking methods (two stability methods were tested for each RF approach). RF methods shared a significant high number of top ranked SNPs (an average of 81 out of 96 SNPs among all applied methods). However, the highest number of shared SNPs was detected between F_ST_ and RF GI 2 methods (87 SNPs, Table 1). Conversely the lowest number of markers (38 SNPs) was detected between PCA and RF GI 1 and/or RF EN 1 (Table 1). More in general, our results suggested as PCA approach identified a different pattern of top ranked SNPs compared to other methods. This might reflect the fact that being an unsupervised technique, PCA simply exploited the observed variability, as already suggested by Schiavo et al. [26]. PCA plots obtained by seven reduced panels (Figure S2) suggested as the identified markers could accurately discriminate native Mediterranean trout ancestry from alien trout. This is in line with what was reported by previous studies where a number of SNPs lower than 100 showed reliable results in individual assignment [25,26,30,47]. Such consideration was also supported by the outcomes of RF analyses applied with the purpose of learning a classification rule to assign specimens to the correct populations through the seven identified panels (Table 2). This is one of the advantages of this machine learning methodology that can be applied for both selection and evaluation purposes. Based on these statistics, all 96 SNP panels performed quite well. The correct prediction proportion in train accuracy for all analysed populations in the reference dataset was 100% for all SNP panels (Table 2). In the test dataset (which included only 20% of the animals of the entire investigated population) a few animals were wrongly classified, but correct prediction proportion (test accuracy) was still high (i.e., ≥92%). In particular, the highest value was observed for the SNP panel derived using the RF GI 2 and RF EN 2 methods (0.97), whereas the lowest for Delta and F_ST_ methods (0.92). Performance outcomes appeared in line with the fact that there was a general high SNPs overlapping between all tested approaches, excluding the Delta and F_ST_ panels, and this supported the idea that most informative markers were effectively selected in our study. This consideration has been also supported by the fact that significant low r^2^ values were estimated between ancestry proportions obtained using the full set of SNP and 1000 random reduced panels (Figure S3); whereas high r^2^ across all panels was detected for both rivers when candidate SNP panels were tested (r^2^ ≥ 0.973; Table 3).

In total, 47 SNPs resulted in common among all seven identified panels and therefore declared as main candidate AIMs. Our results showed that such AIMs can accurately discriminate Mediterranean native trout ancestry from alien as well. In particular, we assessed the performance of such SNPs panel in identifying crosses between native and alien trout using both simulated and real data (Figure 2 and Figure 3; Table S4). As expected, using the AIMs on simulated data performed better than on real admixed trout samples, since simulated individuals were generated from the same reference populations used to select the best AIMs. The mating system applied in simulations generated simplified admixture patterns with respect to those occurring in real populations. Indeed, real admixed populations presented a more complex genetic make-up, influenced by introgression events. Performance outcomes of 47 AIMs on reference population were in line with expectations (train accuracy 100%, test accuracy 92%; Table 2). In addition, r^2^ was quite high (r^2^ was 0.955 and 0.979 for Biferno and Volturno rivers, respectively; Table 3). The lower r^2^ detected in Biferno is consistent with what reported by Palombo et al. [6], which described a more introgressed scenario in such river. Furthermore, the heterogeneous distribution of trout population ancestry scores, obtained through 47 AIMs, within each introgression class, estimated through the combination of mtDNA and LDH-C1* genotypes [33] (Figure S1), suggested that selected AIMs panel could be an effective tool to support conservation and monitoring activities within LIFE17 NAT/IT/000547 Project. Moreover, considering the growing interest in restoring the genetic integrity status of Mediterranean trout populations in Italy, the development of a customized multiplex PCR panel for simple amplicon sequencing may help to confirm our approach outcomes outside Molise rivers.

Noticeably, the distribution of 47 common AIMs along the genome appeared to be heterogeneous, the higher number of identified AIMs were harbored on chromosome 12, 19, and 26 (Figure 4). In this regard, it is interesting to note that this distribution does not reflect the chromosome size, suggesting the possible presence of a selection signature even if no interesting genes were identifiable and no pathways resulted enriched.

## 5. Conclusions

The use of molecular tool to support Brown trout conservation programs and management is of paramount importance; however, conventional molecular markers are often insufficient to classify the specimens at individual level and/or are expensive and time consuming.

In this work, a SNP-array technology and ML approach were combined for the first time to select most informative markers for Atlantic and Mediterranean trout identification. A reduced panel of 47 AIMs was identified. The high correlations between ancestry coefficients calculated using the full set of SNPs and the reduced panel, supported the idea that such panel encompassed AIMs with the high discriminant capacity.

Further studies with larger samples size and/or new populations are required to corroborate our outcomes outside Molise rivers’ basins and to develop a customized multiplex PCR panel to run massive genotyping based on simple amplicon sequencing for Mediterranean tout populations in Italy. However, the methodology described in this study could be useful for the AIMs identification in other species.

## Figures and Tables

**Table 1:** Number of SNPs shared between pairs of SNP panels determined with the seven different methods reported in this study (in the diagonal, the 96 SNPs).

Method	RF GI 1	RF GI 2	RF EN 1	RF EN 2	Delta	F_ST_	PCA
**RF GI 1**	96						
**RF GI 2**	83	96					
**RF EN 1**	89	81	96				
**RF EN 2**	85	89	84	96			
**Delta**	79	77	80	80	96		
**F_ST_**	81	81	80	83	88	96	
**PCA**	52	50	53	53	56	57	96

**Table 2:** Out Of Bag (OOB) and the accuracy classification scores obtained by RF algorithm considering the reference and the test trout populations by using the seven 96 SNP panels.

Method	OOB Score	Train Accuracy	Test Accuracy
RF GI 1	90%	100%	95%
RF GI 2	87%	100%	97%
RF EN 1	85%	100%	95%
RF EN 2	86%	100%	97%
Delta	91%	100%	92%
F_ST_	87%	100%	92%
PCA	87%	100%	95%

**Table 3:** Coefficient of determination values (r^2^) calculated between the ancestry percentages using the full set of SNPs and the AIM panels in case study populations. N is the number of SNPs in each panel.

SNPs Panel	N	Biferno (r^2^)	Volturno (r^2^)
Delta	96	0.982	0.989
F_ST_	96	0.981	0.988
PCA	96	0.973	0.984
RF EN 1	96	0.985	0.985
RF EN 2	96	0.986	0.988
RF GI 1	96	0.985	0.985
RF GI 2	96	0.983	0.987
Candidate AIM	47	0.955	0.979

**Table 4:** List of genes pinpointed by VEP tool within or close (<50 Kbps) to common SNPs included in the panels selected by the seven different methods used in this study (RF GI1, RF GI2, RF EN1, RF EN2, Delta, F_ST_ and PCA).

SNP	Chr	Genomic Position (bp)	Gene(s)
AX-89926492	1	36,478,362	*ENSSTUG00000034565*
AX-89933844	3	56,808,067	*MTX1A, THBS3A, ENSSTUG00000008371, HJV, ITGA8, ENSSTUG00000009416*
AX-89957249	3	49,711,361	*ENSSTUG00000029747*
AX-89933361	4	43,428,599	*CBR4, SH3RF1*
AX-89954271	5	11,245,515	*GFRA1, CCDC172*
AX-89955512	5	31,053,287	*FUOM, ENSSTUG00000037533, ZGC:66426, ERLIN1*
AX-89964745	6	28,034,638	*MRC1A, SLC39A12, CACNB2*
AX-89965418	6	53,152,780	*USP9, ENSSTUG00000025824, DDX3XA, TGDS, GPR180, SLC5A3A, SI:CH211-132G1.7*
AX-89922103	8	11,821,548	*RGS12A, MSANTD1, DTX4A*
AX-89923685	8	40,108,784	*ENSSTUG00000017894, ETV6, ENSSTUG00000017923*
AX-89930404	10	9,847,082	*FMNL1, ENSSTUG00000009061, GRB7*
AX-89926808	12	25,219,858	*CYP2R1, PDE3B*
AX-89935881	12	68,862,681	*MED13L*
AX-89941680	12	72,156,842	*GPSM1B, LHX3*
AX-89943019	12	78,807,143	*MPDU1A, ESRRA, KCNK4, STX5AL, EHD1B*
AX-89944919	12	68,082,269	*ENSSTUG00000031577, TJP2A, ENSSTUG00000036079, SMC5, ZFAND5B*
AX-89966227	12	24,351,329	*POLR2L, DAGLA, EXT2, SYT7B, SDHAF2, CPSF7*
AX-89937326	13	48,590,388	*GRIK4*
AX-89970985	13	27,301,563	*PPP3CB, UBE2D2, ENSSTUG00000035840, ENSSTUG00000035847, PSD2*
AX-89928338	14	30,569,008	*MRPL20, ATAD3A, TMEM240B, SSU72, ORA4, ENSSTUG00000008474, CCNL1B, VWA1*
AX-89965056	14	22,507,646	*GATA2A*
AX-89976571	14	25,409,004	*FHIT*
AX-89975434	15	23,622,069	*IFT46, VPS11, HYOU1, H2AX1, ZPR1*
AX-89971379	16	37,104,371	*PARK7, KCNAB2A*
AX-89961240	19	43,548,455	*TMEM164, AMMECR1, KIF4, MRPS12, ENSSTUG00000016882, ENSSTUG00000016884, FIBPB*
AX-89961754	21	24,970,403	*TMEM53, TESK2, TOE1*
AX-89969654	22	13,715,689	*MYO9B, S1PR4, MIR24-4, ENSSTUG00000015334, ENSSTUG00000015336*
AX-89957356	24	15,818,961	*DOCK9*
AX-89924719	25	31,489,217	*MYCLA, NT5C1AB, ENSSTUG00000048637*
AX-89935421	25	23,270,420	*ODC1, UTP25, ENSSTUG00000028825, ZGC:123321, LAMTOR3, ENSSTUG00000028866, ATP10D*
AX-89950643	25	32,760,767	*RALGAPA2*
AX-89936803	26	26,628,471	*SRPRA, FAM118B, ILVBL, ENSSTUG00000048185, ENSSTUG00000048296, B3GAT1A*
AX-89959464	26	22,256,971	*SLC47A4, SLC47A3, SLC13A5B, SERPINF2B, ENSSTUG00000049175, ENSSTUG00000049189, RPA1*
AX-89948079	27	20,927,232	*IGSF9B, ENSSTUG00000024232, TMEM127, CIAO1, SNRNP200, SLC20A1A*
AX-89961304	28	42,593,898	*ENSSTUG00000021816, ASXL1, PCMTD2A, MYT1A*
AX-89963552	28	24,003,249	*ENSSTUG00000043167, LRRC47, CEP104*
AX-89961685	29	17,322,240	*-*
AX-89965310	33	12,906,283	*ENSSTUG00000029959*
AX-89927784	35	3,818,258	*FNDC1, OTOFA*
AX-89958723	35	4,892,093	*ENSSTUG00000020096, TRMT6, FERMT1, BMP2B*
AX-89938669	38	8,489,802	*RIMS1, KCNQ5B*

## Data Availability

The data presented in this study are available on request from the corresponding author. Supporting data can be made available to bona fide researchers subject to a non-disclosure agreement.

## Supplementary Materials

The following supporting information can be downloaded at: https://www.mdpi.com/article/10.3390/genes13081351/s1, Figure S1: Distribution of trout population ancestry scores for each introgression class, estimated with the combination of mtDNA and *LDH-C1** genotypes according to Pensierini et al. (2006). Figure S2: PCA plots generated using the seven 96-top ranked SNPs panel on entire population split by rivers. Figure S3: Density distribution of coefficients of determination values of 1000 random panels test. The green line represents the correlation value obtained for the GW3 panel. Table S1: Entire dataset encompassing 288 specimens (sample ID) from Biferno and Volturno rivers enrolled in the study with admixture Q scores obtained from 633 SNPs and 47 candidate AIMs panels. *16S* and *LDH* represent genotypes at *16S* rRNA (*16S*) and *LDH-C1** (*LDH*) loci. Classes of introgression (i.e., from class I ‘completely introgressed’ to class VI ‘no introgressed’) were calculated according to Pensierini et al. (2006). Table S2: Lists of top-96 SNPs detected by seven statistical method applied and included in the seven reduced SNP panels. (Rank = position within SNP prioritization hierarchy, RF GI 1 = random forest Gini Index stability occurrence; RF GI 2 = random forest Gini Index stability mean; RF EN 1 = random forest Entropy stability occurrence; RF EN 2 = random forest Entropy stability mean; Delta; F_ST_ = Fixation index; PCA = principal component analysis). Table S3: List of 47 ancestry informative markers (AIMs) resulted in common among all seven SNP panels identified. The numbers refer to the rank position within the hierarchy obtained by seven statistical method applied (RF GI 1 = random forest Gini Index stability occurrence; RF GI 2 = random forest Gini Index stability mean; RF EN 1 = random forest Entropy stability occurrence; RF EN 2 = random forest Entropy stability mean; Delta; F_ST_ = Fixation index; PCA = principal component analysis). Table S4: Out-of-bag (OOB) error scores and correct prediction proportions obtained using random forest classifier with 47 candidate ancestry informative markers (AIMs) and considering both simulated and real hybrid populations split by rivers.
