# Supplementary figures and images for: Identification of Ancestry Informative Markers in Mediterranean Trout Populations of Molise (Italy): A Multi-Methodological Approach with Machine Learning
Source: Genes (Basel). 2022 Jul 28;13(8):1351. doi: 10.3390/genes13081351 (PMC9407066; doi:10.3390/genes13081351)

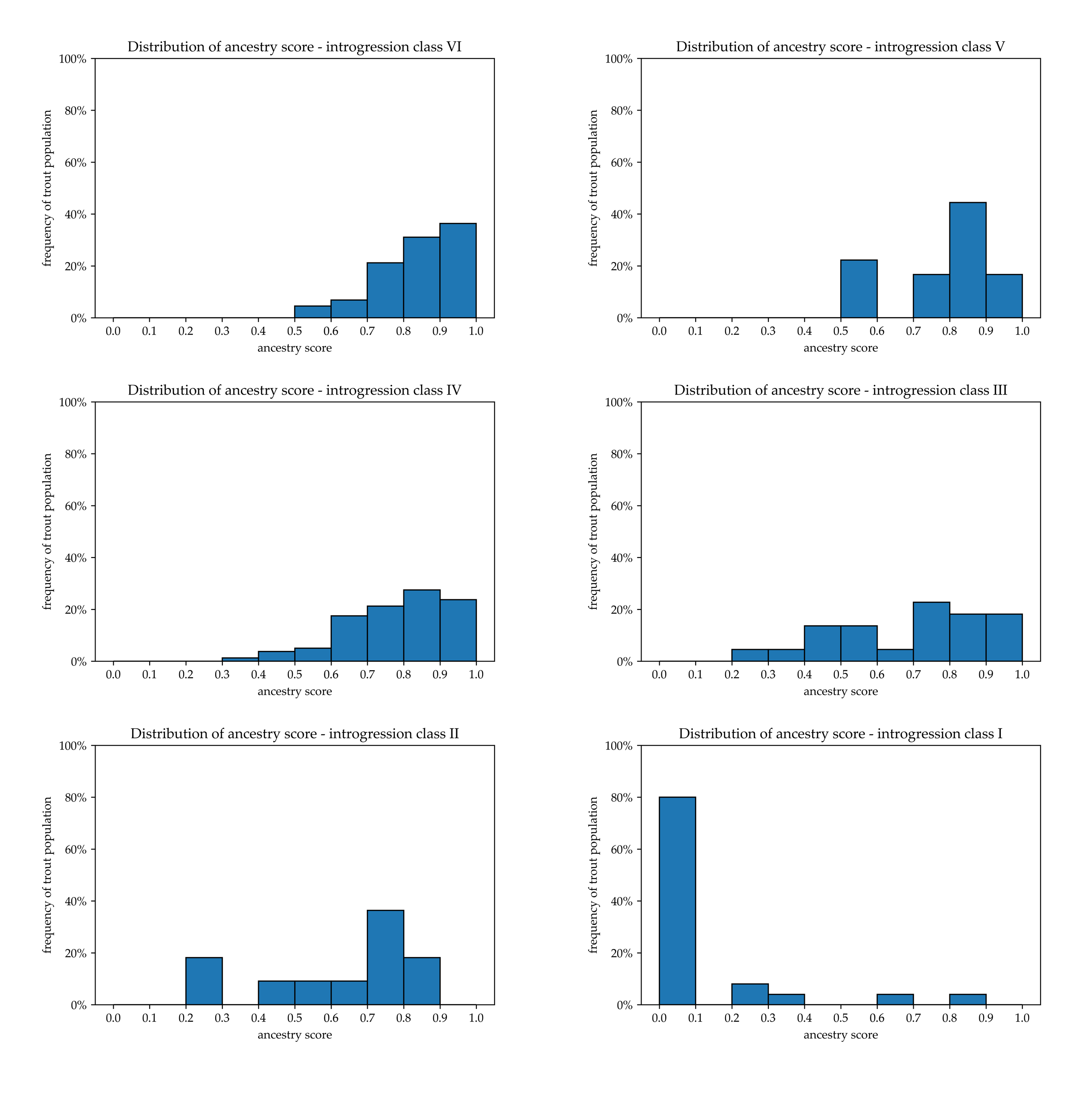

Supplement: Supplementary file 1 [file genes-13-01351-s001.zip › FigureS1.png]

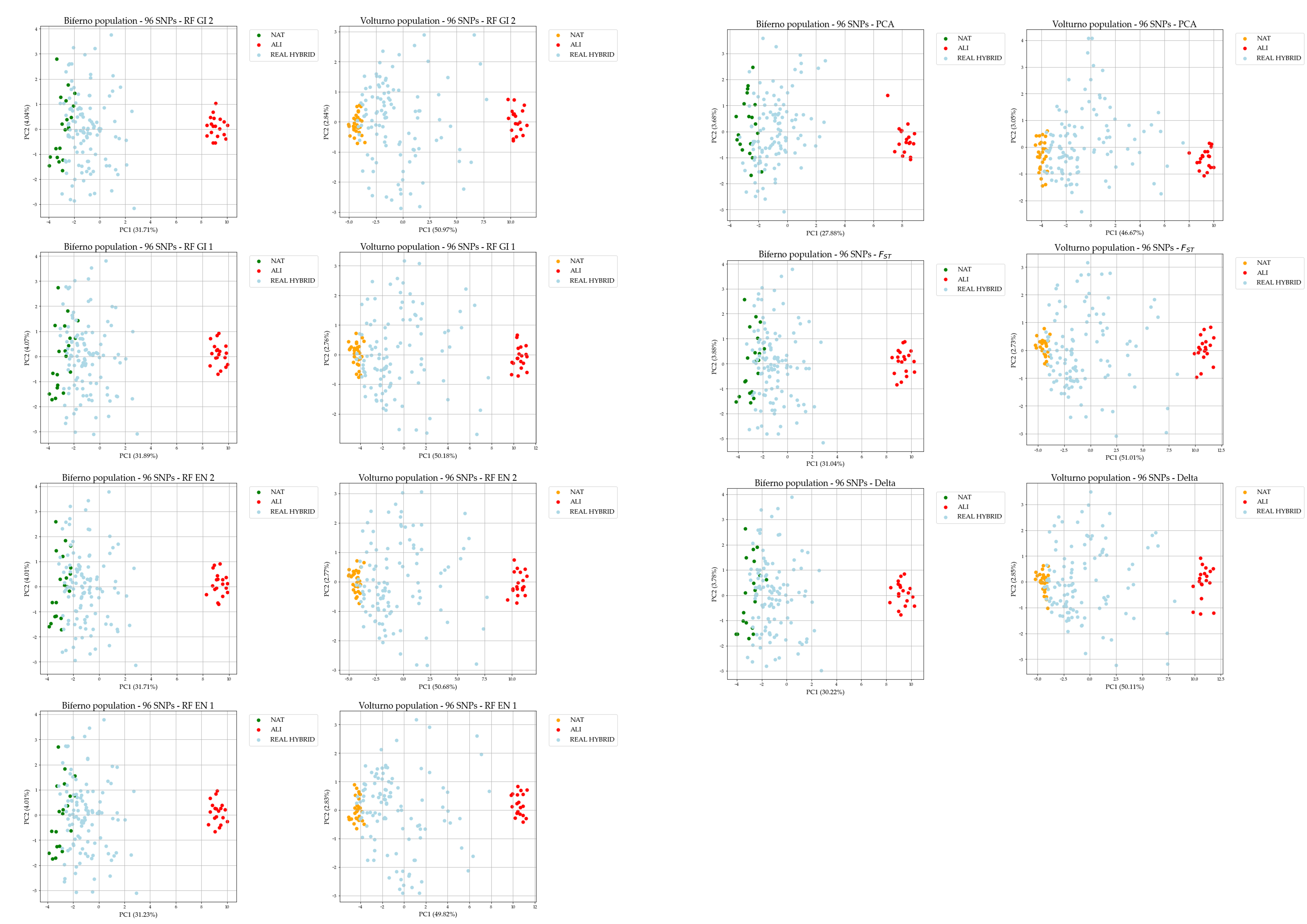

Supplement: Supplementary file 1 [file genes-13-01351-s001.zip › FigureS2.png]

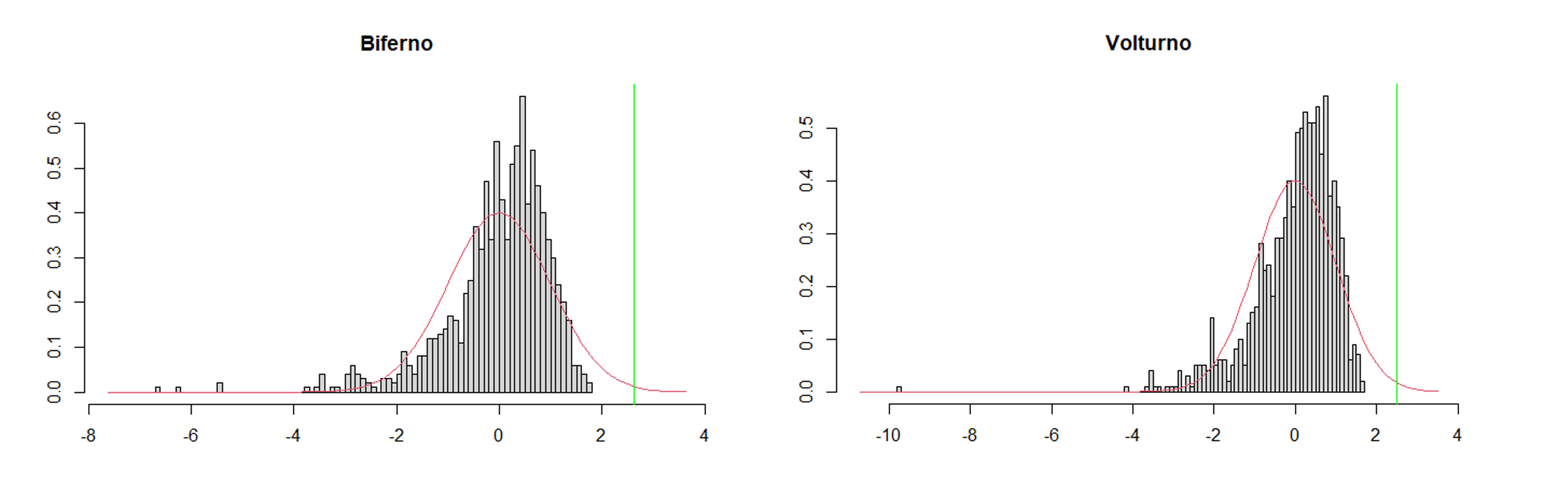

Supplement: Supplementary file 1 [file genes-13-01351-s001.zip › FigureS3.png]
